# ZCCHC3 is a stress granule zinc knuckle protein that strongly suppresses LINE-1 retrotransposition

**DOI:** 10.1371/journal.pgen.1010795

**Published:** 2023-07-05

**Authors:** John L. Goodier, Han Wan, Alisha O. Soares, Laura Sanchez, John Michael Selser, Gavin C. Pereira, Sadik Karma, José Luis García-Pérez, Haig H. Kazazian, Marta M. García Cañadas

**Affiliations:** 1 McKusick-Nathans Department of Genetic Medicine, Johns Hopkins University School of Medicine, Baltimore, Maryland, United States of America; 2 GENYO, Centre for Genomics and Oncological Research: Pfizer, University of Granada, Andalusian Regional Government, Granada, Spain; Fred Hutchinson Cancer Research Center, UNITED STATES

## Abstract

Retrotransposons have generated about half of the human genome and LINE-1s (L1s) are the only autonomously active retrotransposons. The cell has evolved an arsenal of defense mechanisms to protect against retrotransposition with factors we are only beginning to understand. In this study, we investigate Zinc Finger CCHC-Type Containing 3 (ZCCHC3), a gag-like zinc knuckle protein recently reported to function in the innate immune response to infecting viruses. We show that ZCCHC3 also severely restricts human retrotransposons and associates with the L1 ORF1p ribonucleoprotein particle. We identify ZCCHC3 as a *bona fide* stress granule protein, and its association with LINE-1 is further supported by colocalization with L1 ORF1 protein in stress granules, dense cytoplasmic aggregations of proteins and RNAs that contain stalled translation pre-initiation complexes and form when the cell is under stress. Our work also draws links between ZCCHC3 and the anti-viral and retrotransposon restriction factors Mov10 RISC Complex RNA Helicase (MOV10) and Zinc Finger CCCH-Type, Antiviral 1 (ZC3HAV1, also called ZAP). Furthermore, collective evidence from subcellular localization, co-immunoprecipitation, and velocity gradient centrifugation connects ZCCHC3 with the RNA exosome, a multi-subunit ribonuclease complex capable of degrading various species of RNA molecules and that has previously been linked with retrotransposon control.

## Author summary

Retrotransposons are mobile DNA elements that duplicate themselves by a "copy and paste" mechanism using an RNA intermediate. Their misregulation has been linked with some cancers, neuropathologies, and cellular aging. Consequently, the cell has evolved a battery of defenses to protect against retrotransposition. The ancient origin of endogenous retrotransposons suggests their inhibition may have been an evolutionary driver for some host restriction factors, and these may later have been co-opted for defense against exogenous viruses. Zinc Finger CCHC-Type Containing 3, ZCCHC3, protein is a putative host cell restriction factor whose antiviral and innate immune response actions have recently been identified, and that we here show interacts with and limits activity of human retrotransposons in cell culture. In this work we also draw connections between ZCCHC3 and the RNA helicase MOV10 and zinc-finger protein ZAP, host proteins known to restrict viral infection in mammals and previously shown by ourselves and others to potently inhibit human retrotransposition. We also consider that ZCCHC3 may be functionally associated with the cytoplasmic RNA exosome as well as cytoplasmic stress granules, membraneless ribonucleoprotein aggregates that are important for both mRNA metabolism and viral infectivity. Our experiments improve understanding of ZCCHC3 restriction function and may in the future have potential application to the therapeutic treatment of human infectious diseases.

## Introduction

Retrotransposons are mobile DNA elements that duplicate themselves by a "copy and paste" mechanism using an RNA intermediate. Their misregulation has been linked with some cancers, neuropathologies, and cellular aging [1–3]. Intrinsic and innate immunity are the first lines of host defense against retroelements. Intrinsic immunity is an antiviral defense response involving constitutively expressed cellular proteins (host restriction factors), while innate immunity, among many functions, induces interferon (IFN) and IFN-stimulated gene products following recognition of pathogen-associated molecular patterns (PAMPs) [4]. A better understanding of how the immune system keeps both infectious viruses and endogenous retrotransposons in check is critical. Retrotransposons can have negative consequences for the genome and cellular integrity, and a range of mechanisms have evolved to limit their activity (Reviewed in [5, 6]). At least half of human DNA derives from retrotransposition, and approximately 100 non-long terminal repeat (non-LTR) Long Interspersed Element-1 (LINE-1 or L1) retrotransposons are potentially insertionally active in any human individual [7–9]. The six kilobase bicistronic human L1 constitutes 17 percent of the human genome and possesses a 5’ untranslated region (UTR) that functions as an internal promoter, two open reading frames (ORF1 and ORF2), and a 3’ UTR which ends in a poly(A) signal and tail. ORF2 encodes a 150 kilodalton (kD) protein with endonuclease and reverse transcriptase activities. While the 40 kD L1 ORF1 protein (ORF1p) binds RNA and is essential for retrotransposition, its precise role remains unclear [10].

In this study, we investigate the protein ZCCHC3 (Zinc Finger CCHC-Type Containing 3) and its links with LINE-1 retrotransposons. ZCCHC(x) proteins contain a zinc-knuckle or “retroviral zinc-finger” domain and are poorly characterized. (Of interest, L1 ORF2p also contains a gag-like zinc knuckle [11, 12]). When we first began work with ZCCHC3 in 2014, its function was unknown. ZCCHC3, along with ZCCHC7 and ZCCHC9, had been identified as the closest human homologues of yeast nuclear RNA exosome-associated TRAMP1 complex components Air1 and Air2 (37 and 39 percent protein identity, respectively) [12–14]. In an siRNA screen, knock-down (KD) of ZCCHC3 increased infectivity of Hepatitis C RNA virus [15]. The ZCCHC3 gene was also listed as a possible target for miRNA miR-183 [16] and copurified with the HIV gag interactome [17]. In addition, affinity capture and mass spectroscopy (MS) analyses by Taylor et al. [18] found ZCCHC3 to be associated with L1 ORF1p and ORF2p ribonucleoprotein (RNP) complexes.

Then in 2018, a series of articles from Wuhan University revealed ZCCHC3 to be a co-sensor of cyclic GMP-AMP (cGAMP) synthase (cGAS) for the recognition of cytosolic dsDNA; cGAS catalyzes synthesis of the second messenger molecule cGAMP, which in turn binds and activates the adaptor STING to initiate an innate antiviral response. Furthermore, ZCCHC3 was shown to bind dsRNA and act as a positive regulator of RIG-I-like receptor (RLR), including RIG-I (retinoic acid-inducible gene-I) and MDA5, and Toll-like receptor 3 (TLR3) signaling. Significantly, ZCCHC3 KO mice, while showing normal development, had increased lethality following challenge by RNA or DNA viruses, including herpes simplex, vaccinia, encephalomyocarditis, and vesicular stomatitis viruses. It was suggested that ZCCHC3 deficiency inhibits RNA virus-induced transcription of downstream antiviral genes [19–21]. In the same year, Taylor et al. [22] reported inhibitory effects of ZCCHC3 on L1 activity in a cell culture assay for retrotransposition. Since then, ZCCHC3 has been shown to be part of the SARS-CoV-2 virus protein interactome [23, 24] and to inhibit avian H9N2 virus and pseudorabies virus through type I IFN signaling [25, 26].

In a series of cell culture experiments, we confirm here that ZCCHC3 associates with the L1 ORF1 RNP and limits its retrotransposition activity. ZCCHC3 is revealed by immunofluorescence (IF) to be a protein of stress granules (SGs) where it colocalizes closely with ORF1p in cells under stress. We report associations of ZCCHC3 protein with Mov10 RISC Complex RNA Helicase (MOV10) and Zinc Finger CCCH-Type Containing, Antiviral 1 (ZC3HAV1, also known as ZAP or PARP13), two previously identified factors of the immune response against both infecting viruses and endogenous retroelements, including L1 [27–32]. We also link ZCCHC3 with components of the RNA exosome complex, previously shown to be involved in retrotransposon control [33–36].

## Results

### ZCCHC3 protein interacts with itself and the L1 ORF1p RNP

**Fig 1:**
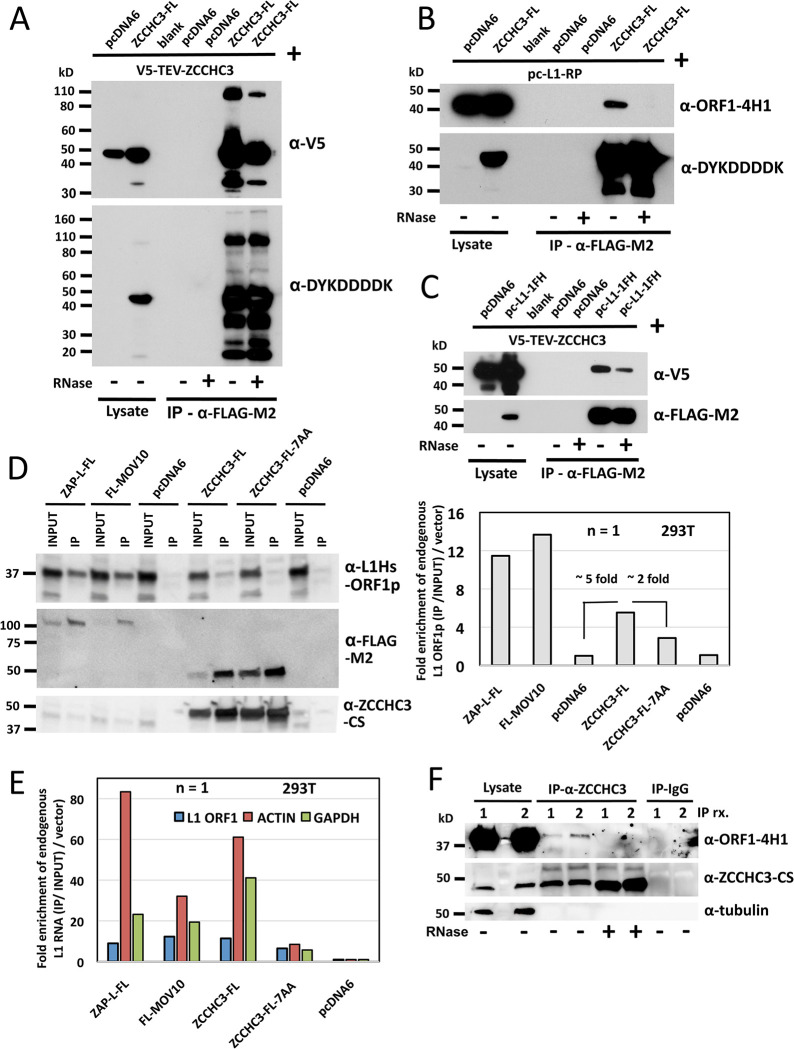
ZCCHC3 protein expressed in 293T cells forms multimers and associates with L1 RNP complexes. (A) ZCCHC3-FL and V5-TEV-ZCCHC3 proteins co-IP on α-FLAG-M2 affinity gel indicating their binding and multimerization. This is further supported by a higher molecular weight band visible at about 95 kD (top panel). In the lower panel we assume bands smaller than the 44 kD full-length ZCCHC3-FL are degradation products occurring during the IP and elution protocol. Vector only control was pcDNA6 myc-his B (pcDNA6). α-DYKDDDDK is an antibody product of Cell Signaling Technology and recognizes FLAG epitope tag. (B) FLAG-tagged ZCCHC3 IPed from HEK 293T cell lysates binds untagged L1 ORF1p complexes coexpressed from a full-length L1 (construct pc-L1-RP). ORF1p was detected by monoclonal α-ORF1-4H1 antibody [38]. Interaction was lost following treatment of lysates with RNase (+, last lane). (C) FLAG-HA-tagged ORF1p expressed from a full-length L1 (pc-L1-1FH) co-IPs V5-TEV-ZCCHC3 complexes from 293T cells. Interaction was diminished by treatment with RNase (+, last lane). (D) FLAG-tagged ZAP, MOV10, and ZCCHC3 co-IP with endogenous L1 ORF1p following affinity purification with α-FLAG-M2 antibody bound to Protein G Dynabeads (left). For this Western we used polyclonal α-L1Hs-ORF1p antibody. The bar chart (right) shows fold-enrichment of bound ORF1p after bands were quantified with ImageJ software (IP/ Input compared with empty vector). Association of ORF1p with the zinc-finger mutant ZCCHC3-FL-7AA was reduced 2-fold compared with wild-type ZCCHC3-FL. Empty vector pcDNA6 was used in duplicate as a negative control. (E) ZAP-L-FL, FL-MOV10, ZCCHC3-FL, and ZCCHC3-FL-7AA co-IP endogenous L1 RNA following affinity purification with α-FLAG-M2 antibody bound to Protein G Dynabeads. RT-qPCR was used to quantitate in the input and IP fractions L1 mRNA (blue bars; detected with the N51 primer pair targeting ORF1 sequence), and as controls β-actin mRNA (red bars) and GAPDH mRNA (green bars). Analyses by real-time RT-qPCR used the Relative Standard Curve Method. For each construct, we calculated the ratio of RNA of the IP fraction/ RNA of the input fraction compared with the empty vector (shown as fold enrichment on the y-axis). Results shown are the average of triplicate RT-qPCR reactions for one IP experiment. (F) α-ZCCHC3-CS antibody bound to Protein G Dynabeads was used in two separate IP reactions [1 and 2] to show that endogenous ZCCHC3 protein binds endogenous L1 ORF1p. Interaction was lost with RNaseA treatment (+, lanes 6 and 7). Normal rabbit IgG linked to Dynabeads was used as negative control and did not bind ORF1p (last two lanes). n = number of biological replicates.

ZCCHC3 protein contains four low complexity domains of unknown function between residues 4 and 142, although the N terminus has been reported to interact with TLR3 adaptor protein TRIF and helicase and caspase recruitment domains (CARDs) of the RIG-I and MDA5 receptors. Three C_2_HC zinc fingers in the C-terminus (residues 335–387) bind dsRNA and DNA and facilitate interaction with TLR3 and binding of dsRNA to RIG-I/MDA5 [20, 21]. Zinc knuckle domains are involved not only in nucleic acid recognition, but also in protein-protein interactions, including homodimerization [37]. We therefore first tested if ZCCHC3 protein bound itself by coexpressing in human embryonic kidney (HEK) 293T cells ZCCHC3 variants tagged with a C-terminal FLAG epitope tag (ZCCHC3-FL) or N-terminal V5-tobacco etch virus cleavage site epitope tag (V5-TEV-ZCCHC3), isolating cell lysates, and performing magnetic affinity gel α-FLAG-M2 immunoprecipitation (IP). Strong co-IP of the expected 47 kD V5-TEV-ZCCHC3 protein by ZCCHC3-FL was seen, with a weaker band at approximately 95 kD suggesting multimerization (Fig 1A)

Affinity capture MS studies by Taylor et al. [18, 22] reported ZCCHC3 as an RNA-dependent interaction partner of immunoprecipitated L1 RNPs, but without confirmation of this by direct protein-protein binding assays. In the present study, we demonstrated that ZCCHC3-FL, cotransfected with a full-length L1 construct without tagged ORFs (pc-L1-RP), co-IPed with the ORF1p RNP (detected by α-ORF1-4H1 antibody [38]). This interaction was lost following pretreatment with RNase enzymes (Fig 1B, +, last lane). Conversely, a full-length L1 construct (pc-L1-1FH) with tandem hemagglutinin (HA)-FLAG tagged ORF1 [28] was able to co-IP V5-TEV-ZCCHC3 protein, an interaction diminished by RNase treatment (Fig 1C, last lane).

We next determined that FLAG-tagged wild-type (WT) ZCCHC3, but not empty vector, is able to IP endogenous ORF1p from 293T cells (Fig 1D, left panels). Binding of endogenous ORF1p to ZCCHC3 protein (a 5-fold increase over empty vector controls, Fig 1D, histogram right) was weaker than to ZAP or MOV10 proteins. We also introduced 3 or 7 mutations to critical residues of the ZCCHC3-FL zinc knuckle domain expected to abrogate function to generate mutant constructs ZCCHC3-FL-3AA and ZCCHC3-FL-7AA, respectively. Binding of ORF1p to ZCCHC3-FL-7AA was reduced 2-fold compared with wild-type ZCCHC3-FL, as determined by ImageJ software quantification of band intensities (Fig 1D, histogram right).

We also used real-time RT-qPCR to test eluants of the above IP reactions for binding of L1, β-actin, and GAPDH RNAs by wild-type ZCCHC3-FL and mutant ZCCHC3-FL-7AA proteins (Fig 1E). L1 RNA was detected by primer pair sequences (N51-Fwd and N51-Rev) located in L1 ORF1 (Fig 1E). Compared with empty vector control, ZAP, MOV10, and ZCCHC3 protein complexes all showed binding of the three RNAs. However, the quantity of L1 ORF1 RNA associated with ZCCHC3-FL-7AA protein was reduced by 50 percent compared to ZCCHC3-FL, similar to its reduced binding of ORF1 protein (Fig 1D, right, and 1E). Binding of all three RNA species was also seen for immunoprecipitated FLAG-tagged MOV10 and ZAP proteins (which might be expected if they exist in a common complex with ZCCHC3 protein, see below). Of note, the ORF1p RNP itself has also been shown to bind many different RNA species, including β-actin and GAPDH [22, 39–42].

Finally, we bound α-ZCCHC3-CS antibody to Protein G Dynabeads to determine in two separate IP reactions [1 and 2] that endogenous ZCCHC3 protein co-IPs endogenous ORF1p in an RNA-dependent manner (Fig 1F). Normal rabbit IgG bound to Dynabeads was used as negative control and showed no binding of ORF1p (last two lanes).

Thus, we confirmed and extended previous studies showing that ZCCHC3 protein multimerizes and is associated with the L1 ORF1p RNP, an association that is at least partly dependent on its zinc knuckle domain.

### ZCCHC3-mediated inhibition of L1 retrotransposition is not strictly dependent on its Zn-knuckle domain

Several variations of the original LINE-1 cell culture retrotransposition assay [43] have been developed (reviewed in [5, 44]). In all of these assays, a reporter gene (aminoglycoside 3’-phosphotransferase, blasticidin S resistance, enhanced green fluorescent protein (EGFP), or luciferase) cassette, interrupted by a backwards intron and inserted in opposite transcriptional orientation into the 3’ UTR of a retrotransposition-competent L1, is expressed only when the L1 transcript is spliced, reverse-transcribed, its cDNA inserted in the genome, and the reporter gene expressed from its own promoter.

**Fig 2:**
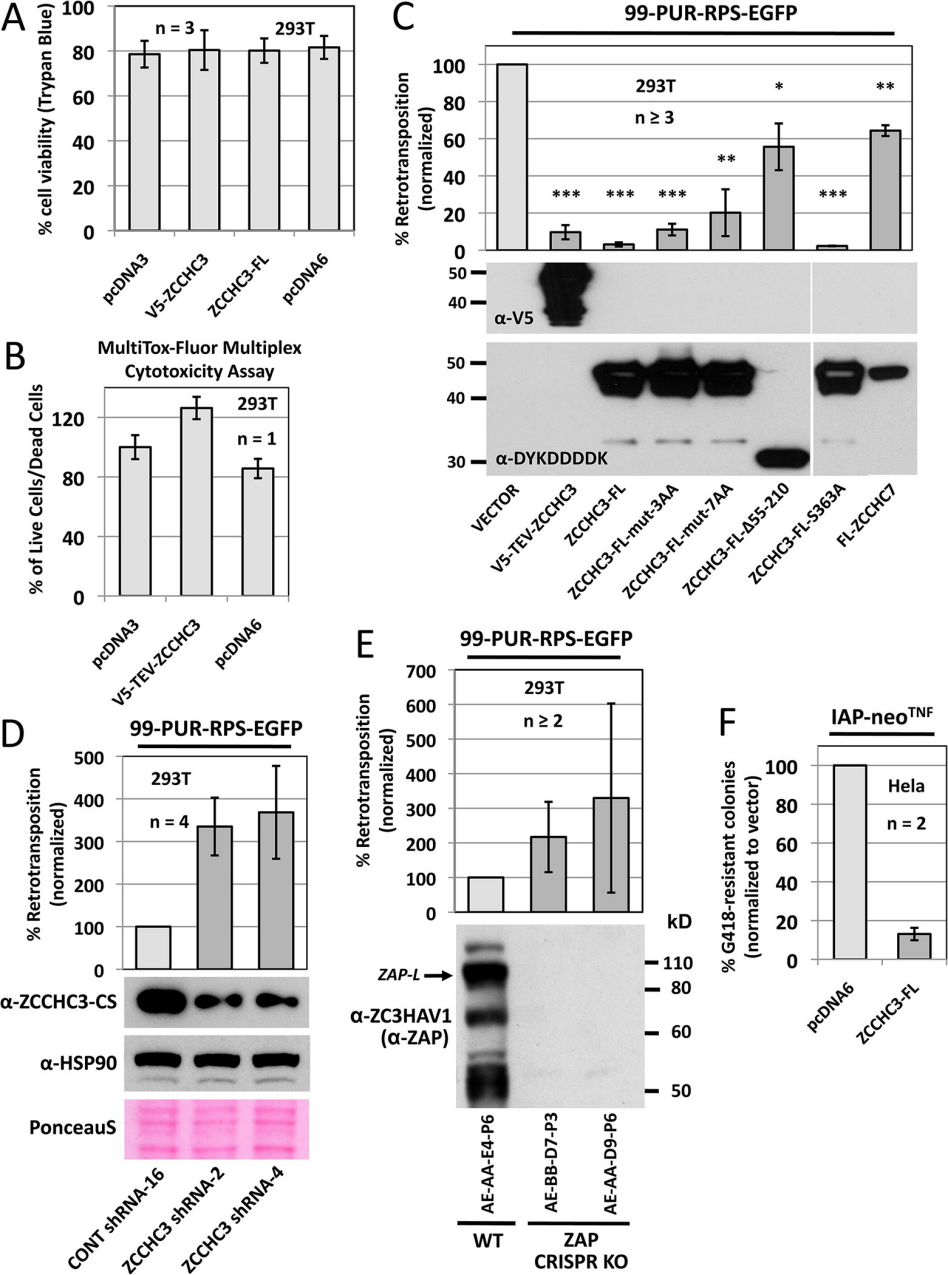
Expression of ZCCHC3 protein is not toxic to cells and alters retrotransposition in cell culture reporter assays. To confirm lack of cytotoxicity due to ZCCHC3 overexpression, 293T cells transfected with tagged ZCCHC3 were (A) stained on day 4 post-transfection with trypan blue and counted using a Cellometer Auto T4 Cell Viability Counter (Nexcelom) or (B) assayed with the MultiTox-Fluor Multiplex Cytotoxicity Assay (Promega). (C) ZCCHC3 exogenous expression inhibits cell culture L1 retrotransposition. The L1 reporter construct 99-PUR-RPS-EGFP [45] was cotransfected in 293T cells with empty vector (pcDNA6 or pcDNA3) or constructs expressing wild-type or mutant ZCCHC3 or ZCCHC7. Five days later, percentages of EGFP-positive cells (i.e., cells with a retrotransposition event) were determined by flow cytometry. Each construct pair was tested in four replicate wells in at least three replicate experiments, and results are normalized to empty vector control (lighter bar). All constructs significantly reduced retrotransposition compared with vector only control (t-test p-values are shown above each bar, *: p<0.05, **: p<0.01, ***: p<0.0001). Bottom panels: Western blots show expression of the tagged test constructs. (D) Loss of endogenous ZCCHC3 expression increases retrotransposition over three-fold. 293T cells were transfected with a scrambled shRNA cloned in vector pLKO.1-TRC (CONT shRNA-16) [46] or shRNA sequences directed against ZCCHC3 [20] and tested for retrotransposition competency of 99-PUR-RPS-EGFP (normalized to empty vector control, lighter bar). Bottom panels: Western blots showing that ZCCHC3 shRNA-2 and ZCCHC3 shRNA-4 strongly decreased endogenous ZCCHC3 protein levels in 293T cells, but had no effect on levels of HSP90. Bottommost panel: following antibody detection, Western transfer blots were stained with Ponceau S as an additional loading control. (E) KO of ZAP expression in 293T cells by CRISPR causes 99-PUR-RPS-EGFP retrotransposition to increase greater than 2-fold. Results are normalized to vector control (lighter bar). Bottom panel: Western blotting shows loss of ZAP protein expression in KO lines. (F) Overexpression of ZCCHC3 strongly inhibits mouse IAP LTR element retrotransposition in HeLa-JVM cells in a neomycin phosphotransferase cell culture reporter assay using vector IAP-neo^TNF^ [48]. The y-axis shows percentage of G418-resistant (retrotransposition-positive) colonies normalized to vector control (lighter bar). n = number of biological replicates.

In 2018 it was reported that overexpression of ZCCHC3 reduced cell culture retrotransposition 90 percent [22]. Since this previous study did not test for possible cytotoxicity of ZCCHC3 overexpression, we first used both trypan-blue exclusion staining and the MultiTox-Fluor Multiplex Cytotoxicity Assay (Promega) to show no toxic effects of overexpression of tagged ZCCHC3 proteins in 293T cells (Fig 2A and 2B).

Then to test for effects on retrotransposition, 293T cells were cotransfected with the 99-PUR-RPS-EGFP retrotransposition reporter construct and tagged ZCCHC3 constructs or empty vector. 99-PUR-RPS-EGFP contains an active human L1 tagged with an EGFP reporter cassette, and GFP-positive retrotransposition events are detected by flow cytometry [45]. Overexpression of V5-TEV-ZCCHC3 or ZCCHC3-FL in cells reduced LINE-1 retrotransposition to 10 and 3 percent of empty vector control, respectively, confirming [22] (Fig 2C). Speculating that the zinc-finger domain may be necessary for restriction of retrotransposition, we tested zinc knuckle mutant constructs ZCCHC3-FL-3AA and ZCCHC3-FL-7AA, but these only modestly increased retrotransposition to 11 and 20 percent of empty vector control, respectively. On the other hand, deletion of an internal N-terminal region (ZCCHC3-FL Δ55–210) increased retrotransposition to 56 percent of vector control. We also altered residue S363 (ZCCHC3-FL-S363A), a serine within the ZCCHC3 zinc-finger domain predicted by our own MS sequencing to be phosphorylated, but found the mutation to have no effect on retrotransposition (Fig 2C). (Other phosphorylated sites predicted by our MS were S66 and S72 with 72 percent protein sequence coverage; ubiquitination of ZCCHC3 was not detected). Overexpression of FLAG-tagged nuclear RNA exosome cofactor ZCCHC7 reduced retrotransposition by only 35 percent of empty vector control (although FL-ZCCHC7 was expressed at a lower level than ZCCHC3-FL, Fig 2C, bottom).

Taylor et al. [22] also found that siRNA KD of ZCCHC3 increased retrotransposition 1.9-fold, although neither sequences of the siRNAs used nor their inhibition efficiencies were reported. We therefore obtained previously characterized ZCCHC3 shRNAs cloned in pSuper-retro vector [20]. In transient transfections, two shRNAs (ZCCHC3 shRNA-2 and ZCCHC3 shRNA-4) strongly reduced levels of endogenous ZCCHC3 protein, and increased retrotransposition approximately 3.5-fold when compared with a scrambled shRNA (CONT shRNA-16) used as control [46] (Fig 2D). During the present study, we also generated a panel of independent ZAP knock-out (KO) clonal 293T cell lines by CRISPR technology and confirmed in two of these greater than 2-fold increase of L1 retrotransposition, similar to that seen for ZCCHC3-depleted cells (Fig 2E; see also below).

Human endogenous retroviruses (HERVs) are LTR retrotransposons that comprise 8% of the human genome and are thought to be incapable of retrotransposition due to inactivating mutations [47]. However, mouse intracisternal A particle (IAP) LTR retrotransposons actively retrotranspose and cause new mutations. Using an established cell culture assay [48], we found that overexpression of ZCCHC3-FL in HeLa-JVM cells [49] reduced retrotransposition activity of an IAP element tagged with a neomycin phosphotransferase reporter cassette to 13 percent of vector only control (Fig 2F).

**Fig 3:**
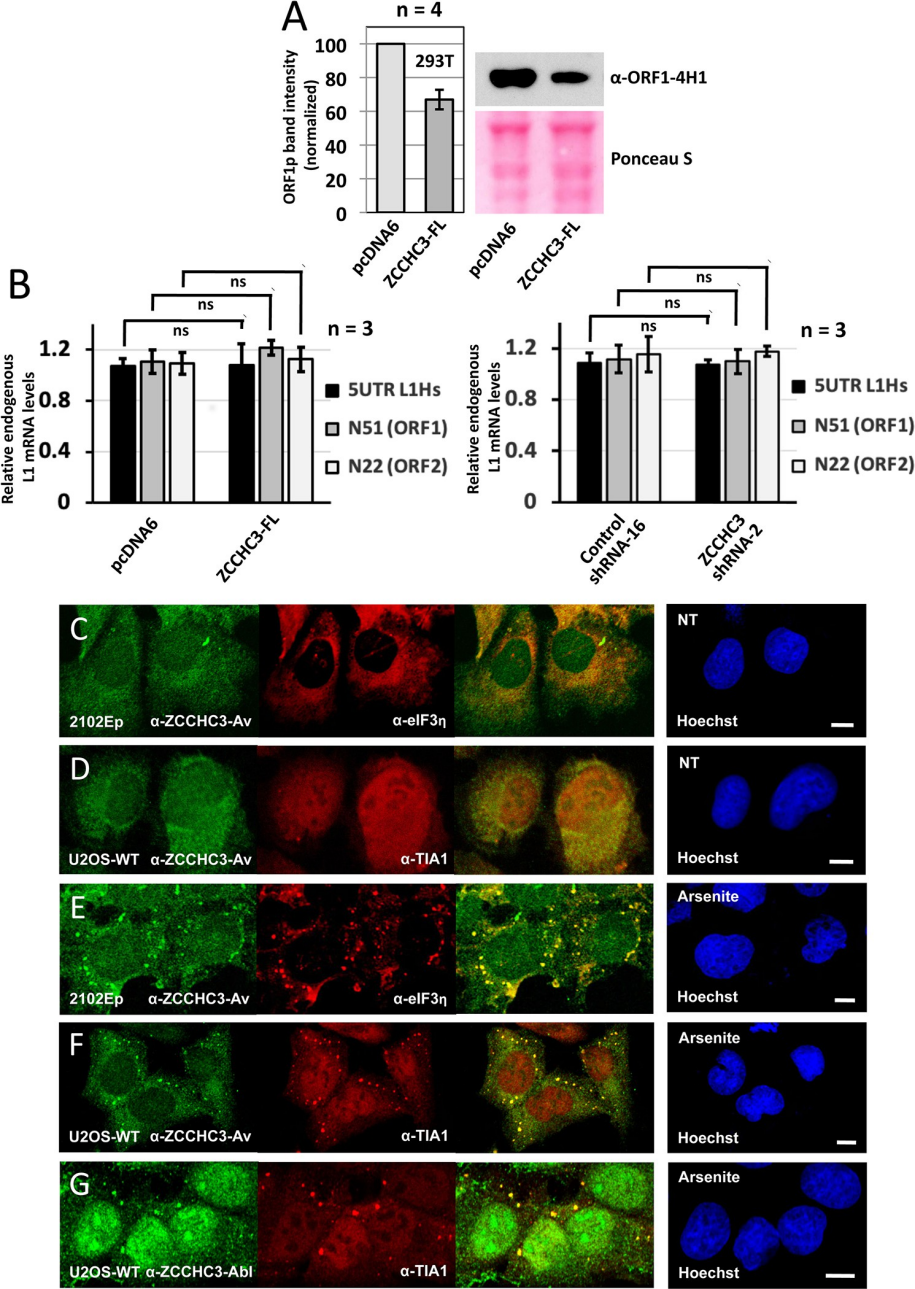
ZCCHC3 protein limits endogenous L1 protein but not L1 RNA expression and co-localizes in stressed cells with markers of cytoplasmic stress granules. (A) Overexpression of ZCCHC3-FL protein significantly (p<0.01) reduces levels of endogenous ORF1p in 293T cells. A sample Western blot from 4 experiments is shown, along with Ponceau S staining of the blot as a loading control (bottom right). Using ImageJ analysis, endogenous ORF1p band intensity was calculated as a percentage of summed Ponceau S-stained bands and normalized to empty vector control (lighter bar, histogram left). n = number of biological replicates. (B) On the other hand, neither overexpression (left) nor shRNA KD (right) of ZCCHC3 protein in 293T cells significantly alters endogenous L1 mRNA levels assayed by RT-qPCR and ΔΔCt analysis. The graph shows L1 RNA levels detected by primer pairs, 5UTR L1Hs (targeting the L1 5’UTR), N51 (targeting ORF1) and N22 (ORF2), relative to the empty vector condition. For each case, GAPDH RNA was used for normalization, and data shows the average of three biological replicates ± s.d. Experimental data were analyzed by two-way ANOVA followed by Sidak´s multiple comparison to calculate statistical significance. ns: not significant. (C and D) Endogenous ZCCHC3 protein, detected by an Aviva Systems Biology antibody (α-ZCCHC3-Av), does not form obvious SGs in untreated 2102Ep or U2OS-WT cells but (E and F) does when cells are stressed with 0.25 mM Na-arsenite. (G) Abclonal α-ZCCHC3-Abl antibody also detects endogenous ZCCHC3 in SGs of arsenite-treated U2OS-WT cells. eIF3η and TIA-1 are canonical SG marker proteins. Cell nuclei were stained with Hoechst 33342 (right-most panels). Size bars are 10 μm.

We next tested if overexpression of ZCCHC3-FL altered levels of endogenous ORF1 protein in 293T cells. Quantitating Western blotting band intensities using ImageJ for four biological replicates, we observed a significant reduction of ORF1p levels (p<0.01) to 66 percent of vector only control (Fig 3A). Finally, using RT-qPCR analyses we determined that neither overexpression (Fig 3B, left histogram) nor shRNA KD (Fig 3B, right histogram) of ZCCHC3 significantly altered levels of endogenous L1 mRNA. Results were consistent for three different primer pairs targeting the L1 5’UTR, ORF1, and ORF2 in three biological replicates. The 5UTR-L1Hs primer pair was designed to preferentially amplify the 5’UTR of young active L1s of the L1Hs (L1PA1) subfamily. Primer pairs N51 and N22 amplify ORF1 and ORF2 sequences, respectively, and have been similarly used in other studies [50–52]. These results suggest that ZCCHC3 protein may inhibit L1 RNPs by means other than RNA degradation.

In summary, in addition to its known antiviral effects, in cell culture ZCCHC3 protein restricts both non-LTR and LTR retrotransposition, an effect that is only partly dependent upon its zinc knuckle domain.

### ZCCHC3 is a bona fide stress granule protein that colocalizes with L1 ORF1p

Early L1 investigations showed ORF1p to be predominantly in the cytoplasm where it forms aggregates [53, 54], and a subset of these aggregates was subsequently identified as stress granules [55, 56]. SGs are discrete cytoplasmic foci that can be induced by a range of stress conditions, including heat shock, osmotic shock, oxidative stress, viral infection, and overexpression of some proteins. They assemble and dissemble rapidly and include the small, but not large, ribosomal subunits bound to translation initiation factors such as eIF2 and eIF3 (reviewed in [57, 58]).

Considering its association with ORF1p, we investigated if ZCCHC3 protein also forms SGs. Using IF and antibody labeling, we observed both endogenous and epitope tagged ZCCHC3 to be evenly distributed without distinct granulation predominantly in the cytoplasm of unstressed cells of multiple lines, including human embryonal carcinoma 2102Ep and osteosarcoma U2OS cells (Fig 3C and 3D). On the other hand, oxidative stress induced by sodium arsenite caused endogenous ZCCHC3 to form large cytoplasmic granules that colocalized with the canonical stress granule marker proteins Eukaryotic Initiation Factor 3, η subunit (eIF3η) and TIA1 Cytotoxic Granule Associated RNA Binding Protein (TIA-1) (Fig 3E and 3F). To be sure that the staining pattern observed was not specific to the Aviva Systems Biology antibody (α-ZCCHC3-Av), we tested a second antibody from ABclonal Technology (α-ZCCHC3-Ab) with similar results (Fig 3G).

**Fig 4:**
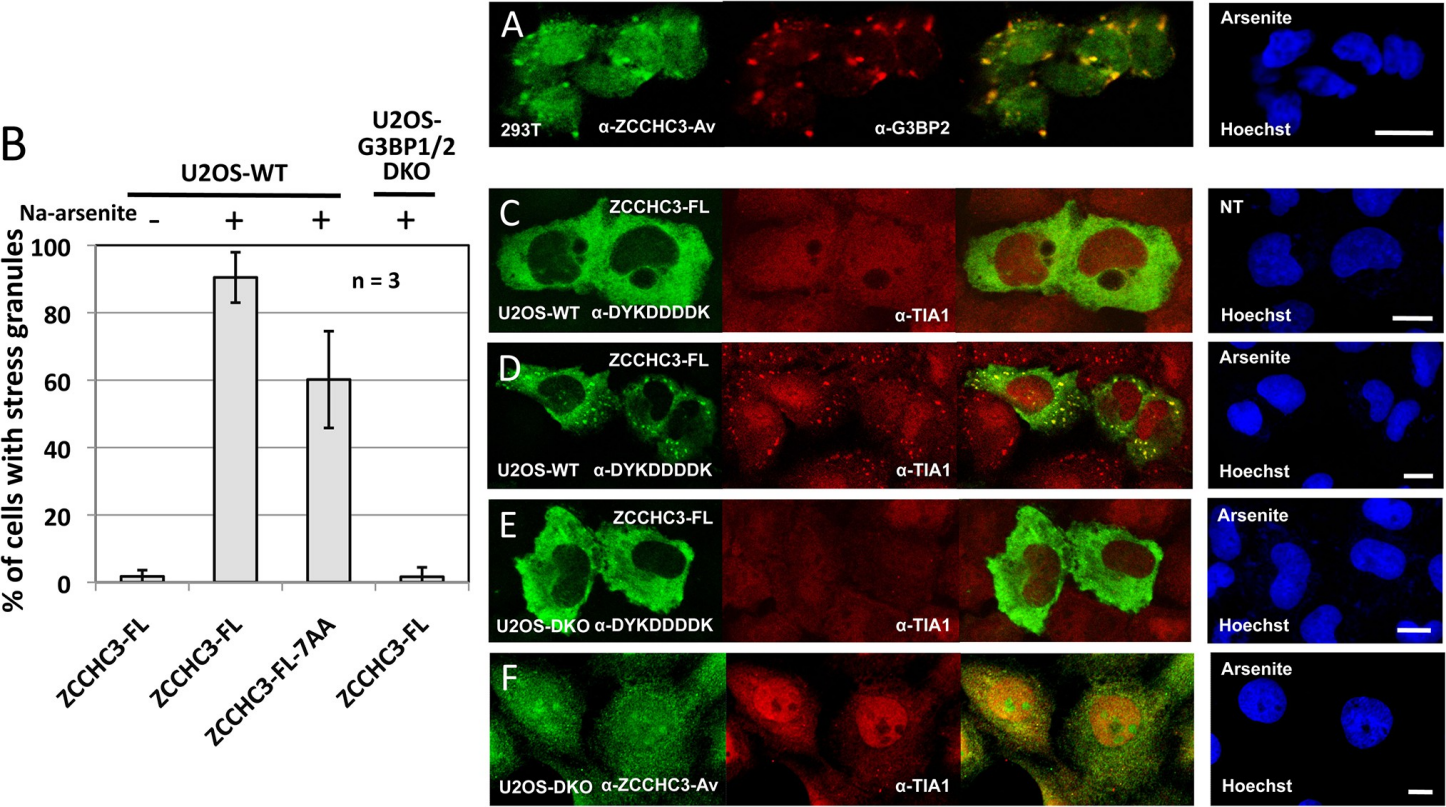
Quantification of cytoplasmic granule formation by tagged ZCCHC3 in wild-type or G3BP1/2 DKO U2OS cell lines. (A) Endogenous ZCCHC3 protein enters SGs marked by endogenous G3BP Stress Granule Assembly Factor 2 (G3BP2) in stressed HEK 293T cells. (B) Summarizing the data of micrograph experiments (C-E), bar graphs were generated by counting at least 200 cells for each transfection. n = number of experimental replicates. (C) FLAG-tagged ZCCHC3 does not form SGs in unstressed U2OS-WT cells, (D) but does when the cells are stressed with Na-arsenite. (E) However, ZCCHC3-FL-containing granules do not form in G3BP1/2 U2OS-DKO cells, even when stressed with Na-arsenite. (F) Similarly, endogenous ZCCHC3 detected by α-ZCCHC3-Av antibody does not form granules in stressed U2OS-DKO cells. NT: no treatment. Cell nuclei were stained with Hoechst 33342 (right-most panels). Size bars are 10 μm.

Endogenous ZCCHC3 also colocalizes in stressed 293T cells with endogenous G3BP Stress Granule Assembly Factor 2 (G3BP2) (Fig 4A). G3BP2 and its homolog G3BP1 are essential for SG assembly under a variety of stress conditions [59, 60]. To confirm that ZCCHC3 enters *bona fide* stress granules, we obtained G3BP1/2 double KO U2OS (U2OS-DKO) cells, along with the parental wild-type line (U2OS-WT) [59], and transfected these with the ZCCHC3-FL construct. Only 1.7 percent of unstressed U2OS-WT cells showed SG formation, but this increased to 90 percent with Na-arsenite treatment (Fig 4B and 4D). Expressing the zinc finger mutant protein ZCCHC3-FL-7AA reduced cytoplasmic granule formation to 60 percent of wild-type ZCCHC3-FL (Fig 4B), a degree of inhibition considerably less than that seen for cell culture retrotransposition (Fig 2C). Notably, in the U2OS-DKO line, granules were observed in only 1.6 percent of cells transfected with WT ZCCHC3-FL, even following Na-arsenite stress (Fig 4B and 4E). Endogenous ZCCHC3 similarly failed to localize to granules in stressed U2OS-DKO cells (Fig 4F). Thus, ZCCHC3 cytoplasmic granules are true SGs.

**Fig 5:**
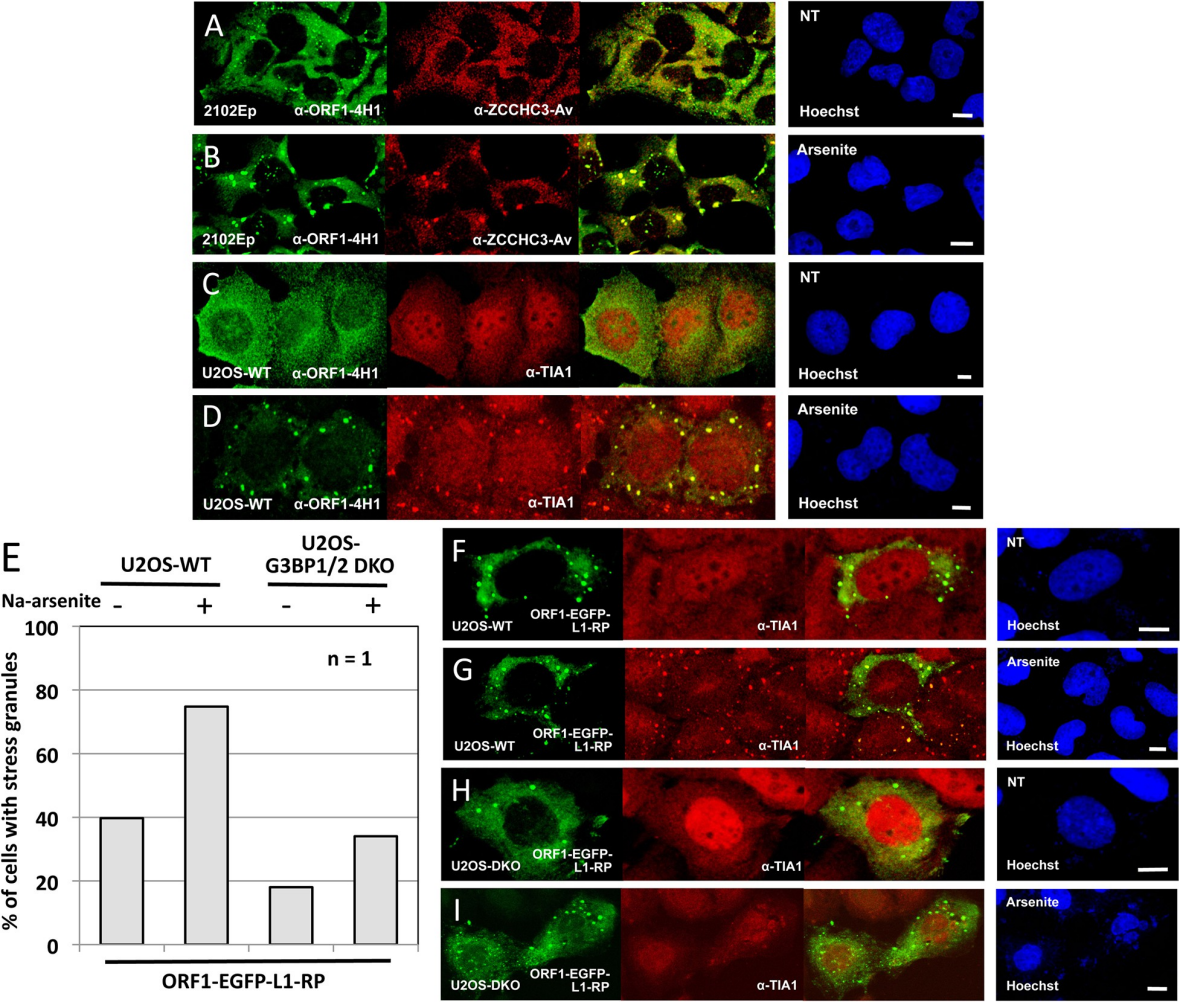
Analysis of cytoplasmic granule formation by ZCCHC3 and L1 ORF1p. (A) Endogenous ORF1p forms granules in untreated, (B) and larger granules in arsenite-stressed 2102Ep cells. However, endogenous ZCCHC3 colocalizes with LINE-1 ORF1 granules in treated 2102Ep cells only (B). (C) However, unlike in untreated 2102Ep cells, endogenous LINE-1 ORF1p does not enter cytoplasmic granules in untreated U2OS-WT cells, (D) but only after Na-arsenite stress. (E) Bar graphs summarize the data of micrograph experiments (F-I). Data were generated by counting at least 200 cells for each transfection. n = number of experimental replicates. GFP-tagged L1 ORF1p expressed from the construct ORF1-EGFP-L1-RP is able to form cytoplasmic granules in both unstressed or stressed (F,G) U2OS-WT or (H,I) U2OS-DKO cells. NT: no treatment. Cell nuclei were stained with Hoechst 33342 (right most panels). Size bars are 10 μm.

We next assayed for colocalization of endogenous ZCCHC3 and L1 ORF1p proteins by IF microscopy. We previously showed that endogenous ORF1p forms small, sometimes numerous cytoplasmic granules in unstressed 293T (not shown) and embryonal carcinoma cells, including nTERA-2 and 2102Ep lines (Fig 5A). Granules increase in size and number upon induced oxidative (by Na-arsenite), mitochondrial (thapsigargin), or osmotic (sorbitol) stress and colocalize with SG markers (Fig 5B) [28, 55, 61]. Unlike ORF1p however, endogenous ZCCHC3 protein does not form distinct granules in unstressed 2102Ep cells (Fig 5A), although the two proteins strongly colocalize in granules under oxidative stress (Fig 5B). It should be noted, as we previously reported, that the ability of endogenous ORF1p to form visible cytoplasmic granules in unstressed cells varies between cell lines [61]. For example, endogenous ORF1p granules form in unstressed 2102Ep (Fig 5A) and nTERA cells, but not obviously in unstressed U2OS-WT cells (Fig 5C); they form only when U2OS-WT cells are stressed (Fig 5D).

We further explored the phenomenon of ORF1p granule formation in unstressed cells by transfecting U2OS-WT and U2OS-DKO cells with ORF1-EGFP-L1-RP, a plasmid with cytomegalovirus promoter, ORF1 C-terminally tagged with EGFP, followed by intact downstream L1 sequence [62]. It is known that overexpression alone of some proteins induces SG formation, and this is the case for plasmid-expressed ORF1p in all cell lines we have tested to date [55, 62, 63]. Unlike the construct ZCCHC3-FL, which shows almost no SGs in unstressed U2OS-WT cells (Fig 4B and 4C), overexpression of ORF1-EGFP-L1-RP induces distinct granules in 40% of the unstressed cells, increasing to 75% when stressed (Fig 5E–5G). Notably, ORF1p-EGFP-L1-RP also forms distinct cytoplasmic granules in many unstressed or stressed U2OS-DKO cells (Fig 5E, 5H and 5I), which ZCCHC3-FL does not (Fig 4B and 4E).

In summary, we find that ZCCHC3 enters SGs and colocalizes with at least a subset of ORF1p cytoplasmic granules in multiple stressed cell lines. While ORF1p enters SGs, it may also concentrate in other unidentified cytoplasmic aggregates that are not SGs (see Discussion). Furthermore, patterns of ORF1p localization vary with cell type.

### Links between ZCCHC3 and L1 controlling factors ZAP and MOV10

The inhibitory action of ZCCHC3 is analogous in a number of ways to the retroelement inhibitory factors MOV10 DExD-box RNA helicase and ZAP zinc-finger protein. These host proteins are known to restrict viral infection in mammals and were shown by ourselves and others to reduce human cell culture retrotransposition by greater than 95 percent when overexpressed or to increase retrotransposition 2- to 3-fold when inhibited (Fig 2E) [28–32, 64].

**Fig 6:**
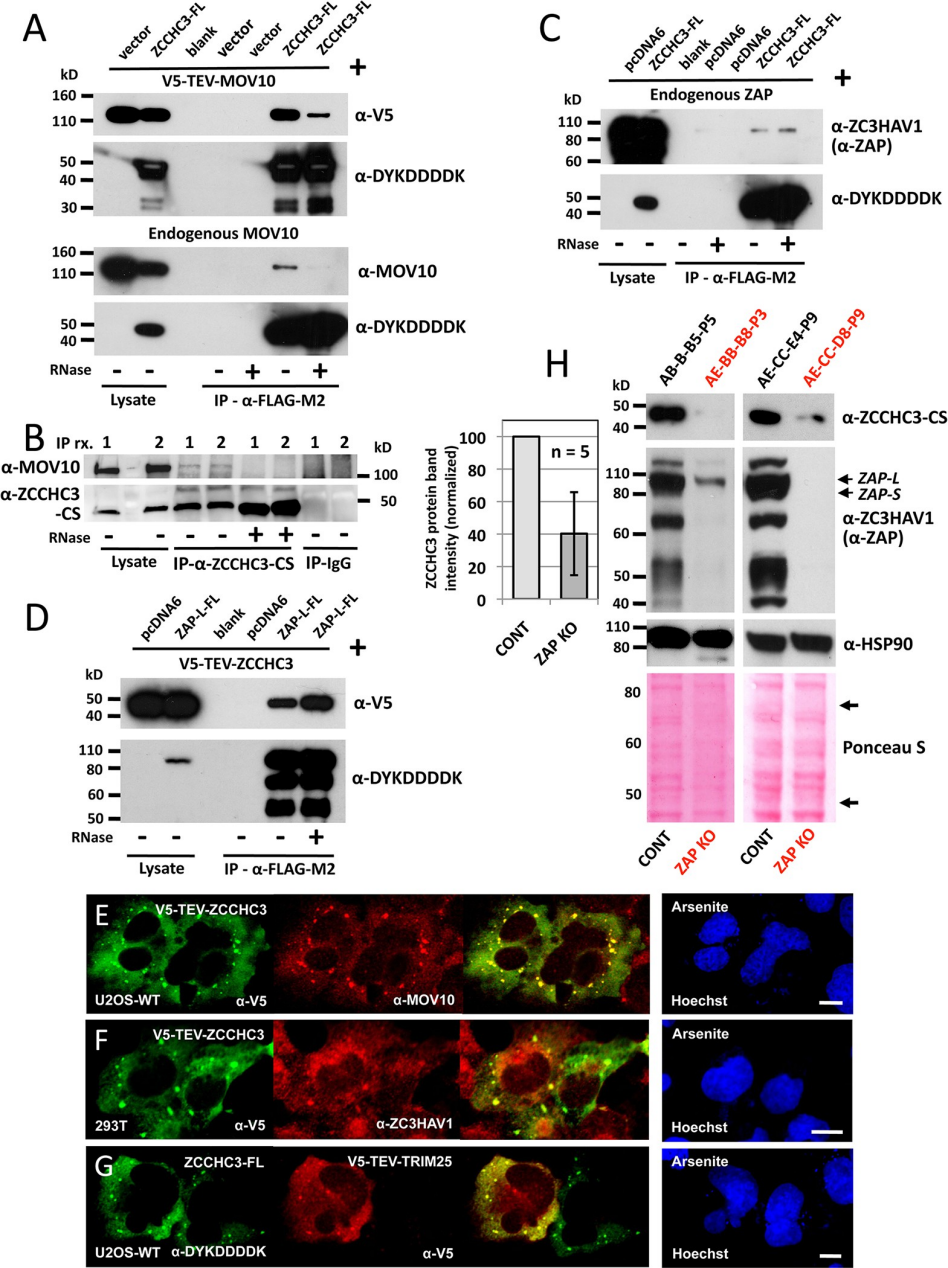
ZCCHC3 protein associates with MOV10 and ZAP proteins. (A) Immunoprecipitated FLAG-tagged ZCCHC3 protein associates with both exogenously expressed (top panels) and endogenous (bottom panels) MOV10 protein in an RNA-dependent manner (last two lanes, -/+ RNaseA treatment). Control vectors were pcDNA5 FRT/TO (top panels) and pcDNA6 myc/his B (bottom panels). (B) α-ZCCHC3-CS antibody bound to Protein G Dynabeads was used in two separate IP experiments [1 and 2] to show that immunoprecipitated endogenous ZCCHC3 protein binds endogenous MOV10 protein and that this interaction is lost with RNaseA treatment (lanes 6 and 7). Normal rabbit IgG was used as negative control and showed no binding of endogenous MOV10 protein (last 2 lanes). (C) FLAG-tagged ZCCHC3 IPs endogenous ZAP protein in an RNA-independent manner (last two lanes, -/+ RNaseA treatment). To generate this data, the Western blot of Fig 6A (lower panel) was stripped. (D) FLAG-tagged ZAP-L long isoform (PARP13.1) co-IPs V5-TEV-ZCCHC3 protein in an RNA-independent manner (last two lanes, -/+). Experiments in A-D were performed in 293T cells. (E and F) Tagged V5-TEV-ZCCHC3 colocalizes with endogenous MOV10 and ZAP proteins in cells stressed with Na-arsenite. (G) Exogenously expressed ZCCHC3-FL and V5-TEV-TRIM25 proteins colocalize in cytoplasmic granules of stressed U2OS-WT cells. (H) Multiple 293T cell lines deleted for ZAP expression by CRISPR protocol (in red text, KO cell lines AE-BB-B8-P3 and AE-CC-D8-P9 are shown as examples) have diminished expression of endogenous ZCCHC3 compared with control lines (top panels). Control lines (ex., AB-B-B5-P5 and AE-CC-E4-P9) were similarly subjected to CRISPR treatment but without successful KO of ZAP expression. Full-length (ZAP-L) and the short (ZAP-S) isoforms of ZAP are indicated by arrows on the Western blot. Reprobing with α-HSP90 antibody and staining of Western transfer blots with Ponceau S (bottom panels) are controls for equal sample loading. Bands were quantitated by ImageJ software for 5 ZAP KO and 5 control cell lines. For each pair of cell lines (KO and control), endogenous ZCCHC3 band intensity was calculated as a percentage of summed Ponceau S-stained bands between the two arrows indicated (bottom right panel) and normalized to empty vector control (histogram, left).

We and others also previously showed that ZAP protein binds in co-IP experiments and colocalizes in IF experiments with both L1 ORF1p and MOV10 proteins [28, 29, 31]. We now demonstrate that tagged or endogenous ZCCHC3 and MOV10 co-IP from 293T cells, an association that is dependent upon RNA (Fig 6A and 6B). Two other studies also identified MOV10 as an interacting partner of ZCCHC3 by quantitative MS [65, 66]. Similarly, FLAG-tagged ZCCHC3 co-IPs with endogenous ZAP (Fig 6C), and FLAG-tagged ZAP co-IPs with V5-TEV-ZCCHC3, in each case independently of RNA (Fig 6D).

IF shows that V5-TEV-ZCCHC3 closely colocalizes with both endogenous MOV10 and ZAP proteins in cytoplasmic granules of cells treated with Na-arsenite (Fig 6E and 6F). We also confirmed that tagged ZCCHC3 and TRIM25 E3 ubiquitin ligase colocalize in granules of U2OS WT cells (Fig 6G): it was recently shown that TRIM25 binds SG proteins G3BP1/2 and ZCCHC3, and that ZCCHC3 recruits TRIM25 to pattern recognition receptor RIG-I and MDA5 complexes to facilitate their polyubiquitination and activation [20, 67, 68]. It has also been reported that TRIM25 is an essential cofactor of ZAP activity and effector of RIG-I and IFN-induced antiviral restriction, including against SARS-CoV-2 [69–74].

We next assessed by Western blotting expression of endogenous ZCCHC3 in our ZAP deficient 293T CRISPR KO clonal cell lines compared with lines that had been similarly subjected to CRISPR treatment, but which failed to show diminished ZAP protein. In all of five ZAP KO lines examined, we saw significant (p<0.01) reduction in ZCCHC3 protein levels (by 60%) compared with their five non-KO controls (Fig 6H). One interpretation of this phenomenon is that ZCCHC3 is stabilized and protected from degradation when bound in complex with ZAP. (This would be analogous to the mutually increased stability in complex shown by amyotrophic lateral sclerosis-related proteins SMCR8 and C9ORF72, as previously reported by ourselves and others [75]). Collectively these data link ZCCHC3 and ZAP.

Thus, it is possible that ZCCHC3, ZAP, TRIM25, and perhaps MOV10 function together in a common complex. Of interest, all four proteins plus G3BP1 were recently reported to be part of the SARS-CoV-2 nucleocapsid protein interactome [23].

### ZCCHC3 associates with the RNA exosome

The RNA exosome is a multi-protein complex present in both the nucleus and cytoplasm that degrades various species of RNAs [76]. It consists of a ring structure core of six RNase PH-like subunits, EXOSC4/RRP41 (human ortholog of yeast Rrp41), EXOSC5/RRP46, EXOSC6/MTR3, EXOSC7/RRP42, EXOSC8/RRP43, and EXOSC9/RRP45), and a cap structure consisting of three S1/KH RNA binding domain subunits (EXOSC1/CSL4, EXOSC2/RRP4, and EXOSC3/RRP40). Two catalytic subunits with endoribonuclease/exoribonuclease activities, EXOSC10/RRP6 and DIS3/RRP44/EXOSC11, or its cytoplasmic homolog DIS3L (also called DIS3L1), associate with the nine-subunit core. Additional targeting complexes also interact with the exosome, including the nuclear exosome targeting (NEXT) complex, which mediates degradation of non-coding RNAs, the predominantly nucleolar Trf4/Air2/Mtr4p Polyadenylation (TRAMP) complex, involved in 3’ end processing and degradation of ribosomal RNAs and snoRNAs, and the cytoplasmic superkiller (SKI) complex, which assists in 3′ to 5′ degradation of mRNA transcripts and has recently been implicated as a host target for several viruses, including SARS-CoV-2 [77, 78].

**Fig 7:**
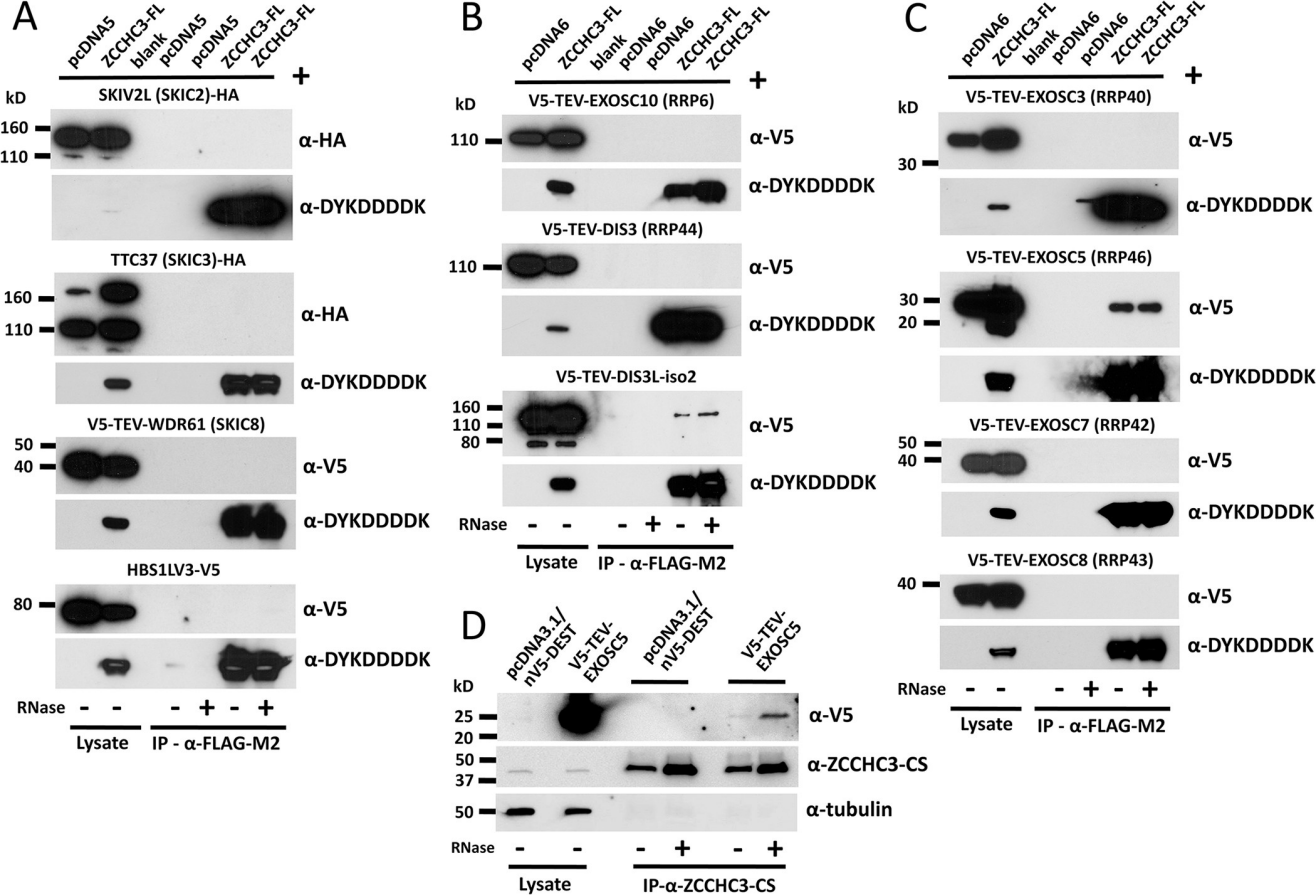
FLAG-tagged ZCCHC3 co-IPs coexpressed tagged EXOSC5 and DIS3L exosome subunits but not other selected (A) cytoplasmic SKI complex components (SKIV2L, TTC37, WDR61, and HBS1LV3), (B) exosome catalytic subunits (EXOSC10, DIS3, and DIS3L), or (C) subunits of the exosome core (EXOSC3, EXOSC5, EXOSC7, and EXOSC8). In (A), the HBS1L short splicing isoform HBS1LV3 reportedly links the SKI complex and cytoplasmic exosome in humans (the canonical HBS1LV1 variant does not associate with the exosome) [83]. In (B), the DIS3L isoform 2 used differs from the canonical sequence by lacking the first 83 amino acids (Acc. # NP_001310865.1). The control empty vectors were pcDNA5 in (A) and pcDNA6 in (B and C). (D) Endogenous ZCCHC3 binds coexpressed V5-TEV-EXOSC5 when immunoprecipitated by α-ZCCHC3-CS antibody bound to Protein G Dynabeads. The control empty vector used was pcDNA3.1/nV5-DEST. The amount of immunoprecipitated EXOSC5 protein increased with RNase treatment (+, last lane). Alpha-tubulin is shown as a loading control (bottom panel). All experiments were performed in HEK 293T cells.

ZCCHC3 (along with ZCCHC7 and ZCCHC9) is a close human homologue of yeast TRAMP complex components Air1 and Air2 [12–14]. Nuclear ZCCHC8 is a component of NEXT, while nucleolar ZCCHC7 is associated with TRAMP and has antiviral properties (and, as we noted above, modest inhibitory effect on cell culture retrotransposition when overexpressed (Fig 2C)) [79–82]. We thus speculated that by analogy ZCCHC3 might be an accessory subunit of the cytoplasmic exosome, and specifically its SKI complex cofactor. Therefore, we performed direct co-IP from 293T cells of ZCCHC3-FL and epitope tagged SKI complex components SKI2 Subunit of Superkiller Complex (SKIV2L/SKIC2), SKI3 Subunit of Superkiller Complex (TTC37/SKIC3), SKI8 Subunit of Superkiller Complex (WDR61/SKIC8), and HBS1 Like Translational GTPase short splicing isoform (HBS1LV3), the latter being the putative human equivalent of yeast Ski7, which links SKI with the exosome [83]. However, no direct interaction with tagged ZCCHC3 protein was detected for any of these proteins (Fig 7A).

We next tested for by co-IP for interaction of tagged ZCCHC3 and exosome catalytic subunits EXOSC10 and nuclear DIS3 (EXOSC11) and its cytoplasmic homolog DIS3L. No interaction with EXOSC10 or DIS3 was detected, but weak RNA-independent binding was seen for cytoplasmic DIS3L isoform 2 (Fig 7B). (Note, the sequence of the alternatively spliced isoform used here differs from full-length DIS3L sequence by missing the first 83 amino acids).

Finally, we tested for binding of ZCCHC3-FL with selected subunits of the 9-subunit exosome core, including EXOSC3, EXOSC5, EXOSC7, and EXOSC8, all tagged on their N-termini with V5-TEV (Fig 7C). Following co-IP from 293T cells, only V5-TEV-EXOSC5 bound ZCCHC3-FL, and this interaction was resistant to treatment with RNase. Furthermore, we showed that α-ZCCHC3-CS antibody bound to Protein G Dynabeads co-IPs endogenous ZCCHC3 together with coexpressed V5-TEV-EXOSC5; this association increased with RNase treatment (Fig 7D). Interestingly, EXOSC5 has been shown by co-localization and proximity ligation assay to interact with cytoplasmic exosome SKI complex component SKIV2L [84]; therefore, perhaps detecting in vitro binding of ZCCHC3 with the SKI complex requires coexpression of EXOSC5, a possibility we did not test.

ZAP similarly associates with the exosome and EXOSC5. Although the Gao lab reported that rat but not human ZAP binds EXOSC5 [85, 86], we previously found that human ZAP-L long isoform (also called PARP13.1), like ZCCHC3, co-IPs with EXOSC5 in the presence or absence of RNase [29]. (Of note, Zhu et al. [86] reported that EXOSC7 also interacts with ZAP, but we found it not to bind with ZCCHC3 (Fig 7C)).

**Fig 8:**
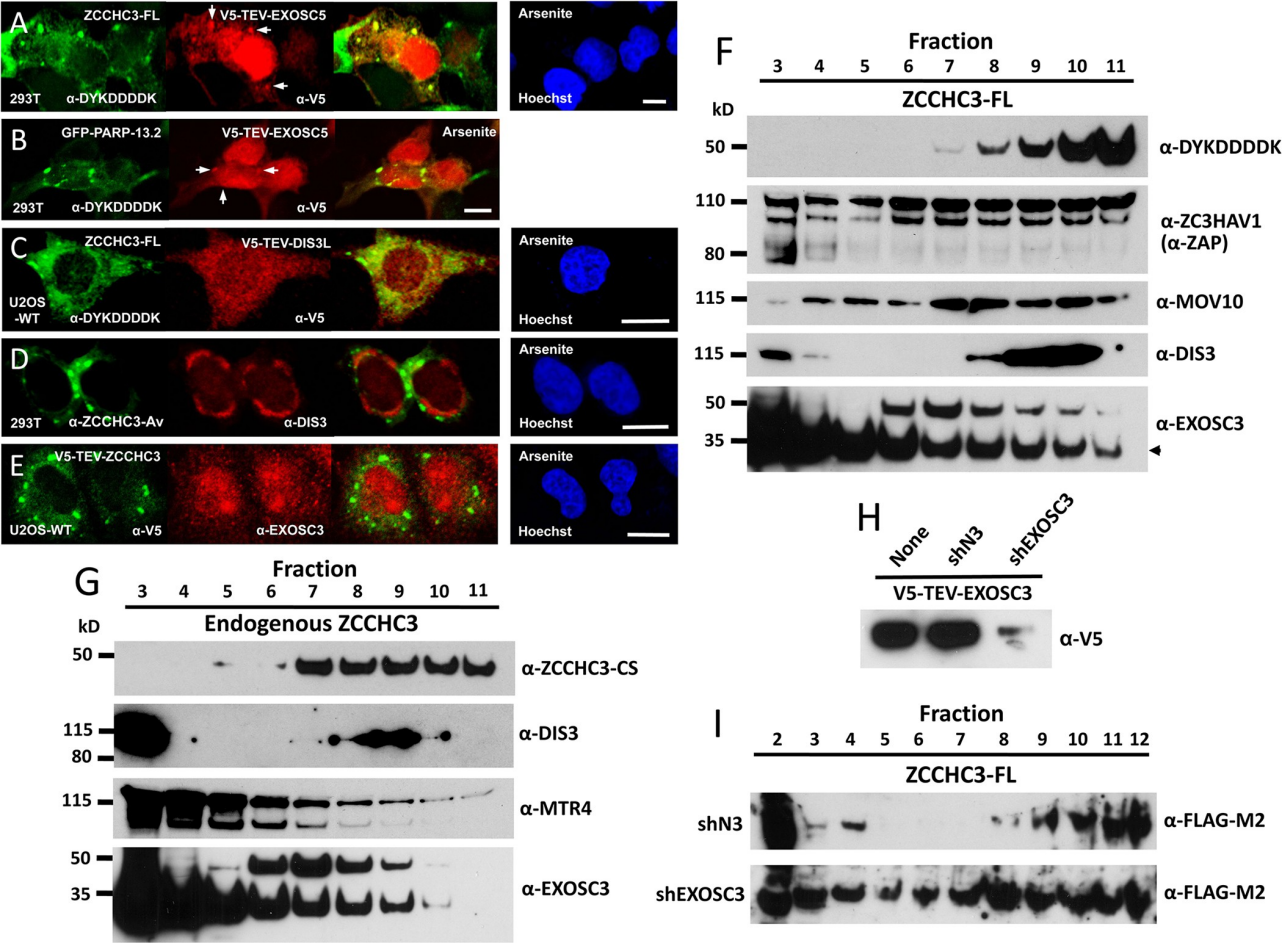
ZCCHC3 protein colocalizes in SGs with exosome component EXOSC5 and cosediments in a sucrose gradient with selected proteins of the exosome as well as MOV10 and ZAP. (A,B) Both ZCCHC3-FL and the short ZAP isoform tagged with GFP (GFP-PARP-13.2) colocalize with tagged exosome component EXOSC5 in cytoplasmic granules of stressed 293T cells (see arrows). C) On the other hand, V5-TEV-DIS3L protein does not colocalize with ZCCHC3-FL in cytoplasmic granules of stressed U2OS-WT cells, (D) nor does endogenous ZCCHC3 protein colocalize with endogenous DIS3L nuclear homolog DIS3 in granules of 293T cells. (E) V5-TEV-ZCCHC3 does not colocalize with endogenous EXOSC3 in SGs of stressed 2U2OS-WT cells. Cell nuclei were stained with Hoechst 33342 (right-most panels). Size bars are 10 μm. (F) HEK 293T cells expressing ZCCHC3-FL were lysed, and lysates were fractionated by sucrose velocity gradient centrifugation. Aliquots of each 900 μl fraction were subjected to SDS-PAGE and Western blotting with antibodies against endogenous MOV10, ZAP, and exosome components. The ~42 kD band above the expected 30 kD EXOSC3 band (marked by an arrow, lower panel) appears more in HMW fractions and may reflect post-translational modification, possibly ubiquitination [123]. (G) Untransfected HEK 293T cell lysates were analyzed as in (F). (H) In 293T cells, protein expression from the V5-TEV-EXOSC3 construct is strongly inhibited by cotransfection of the shEXOSC3 shRNA construct, but not in untransfected or scrambled shRNA (shN3)-transfected controls [34]. (I) HEK 293T cells expressing ZCCHC3-FL and shN3 control (top) or shEXOSC3 (bottom) shRNA constructs were lysed and subjected to sucrose velocity gradient centrifugation as above and analyzed by α-FLAG-M2 antibody. Cotransfection of shEXOSC3 caused redistribution of ZCCHC3 protein throughout the gradient suggesting its dissociation from HMW complexes.

We next tested by IF for protein association. FLAG-tagged ZCCHC3 colocalizes with V5-TEV-EXOSC5 in cytoplasmic granules of cells stressed by Na-arsenite (Fig 8A). GFP-tagged ZAP-S (PARP13.2, a shortened ZAP isoform lacking the PARP domain) similarly colocalizes with V5-TEV-EXOSC5 (Fig 8B). Neither DIS3L, DIS3, nor EXOSC3 colocalized in granules with ZCCHC3 in wild-type U2OS or 293T cells treated with Na-arsenite (Fig 8C–8E).

It was previously reported that ZAP cosediments on centrifugation gradients with endogenous components of the exosome, including EXOSC5 [85]. To provide further evidence of ZCCHC3 association with the exosome, we prepared 293T whole cell extracts alone or transfected with ZCCHC3-FL, fractionated these on discontinuous sucrose gradients by velocity sedimentation centrifugation, removed aliquots, and tested them by Western blotting. Both epitope tagged and endogenous ZCCHC3 proteins concentrated in the lower half of the column in fractions 7 to 11, suggesting they are in high molecular weight (HMW) complexes (Fig 8F and 8G). MOV10 and ZAP overlapped the same HMW fractions as ZCCHC3-FL, although ZAP protein was more distributed across the gradient, as previously reported [85] (Fig 8F). Significantly, exosome catalytic subunit DIS3 displayed a sedimentation profile highly similar to both tagged and endogenous ZCCHC3 with peak levels centered about fraction 9 (Fig 8F and 8G). This was not the case for endogenous ZCCHC3 and Mtr4 exosome RNA helicase (MTR4/SKIV2L2), a subunit of the nuclear exosome targeting NEXT and TRAMP complexes; MTR4 was predominantly in lower molecular weight (LMW) fractions (Fig 8G). Partial overlap of EXOSC3 with both exogenous and endogenous ZCCHC3 proteins was observed (mainly in fractions 7 to 9) although the majority of EXOSC3 protein remained concentrated in LMW fractions (Fig 8F and 8G). EXOSC3 is reportedly less stably associated with the core exosome, and so perhaps dissociates easily in a gradient [87].

It is known that depletion of EXOSC3 destabilizes other exosome subunits in complex, including the catalytic subunits EXOSC10 and DIS3L, causing significant reduction of exosome activity [34, 88]. Therefore, we obtained scrambled shRNA control (shN3) and EXOSC3-targeting shRNA (shEXOSC3) plasmids [34]. We first confirmed that shEXOSC3 reduced levels of cotransfected V5-TEV-EXOSC3 protein by greater than 90 percent compared with untransfected or shN3-transfected controls (Fig 8H). We next prepared lysates of 293T cells cotransfected with ZCCHC3-FL and either shN3 or shEXOSC3, loaded these on sucrose gradients, and again performed centrifugation. Confirming the experiment above, ZCCHC3-FL in the presence of the shN3 negative control was concentrated in HMW fractions 9 and greater (Fig 8I, top panel). In contrast, KD of the exosome by shEXOSC3 caused ZCCHC3-FL protein to shift to LMW fractions, indicating its dissociation from HMW complex, presumably the exosome (Fig 8I, bottom panel).

Together, these data draw links between ZCCHC3 protein and the RNA exosome. Mutual binding and colocalization with EXOSC5 further connect ZAP and ZCCHC3. It has been proposed that ZAP binds EXOSC5 to recruit the 3’-5’ exosome to degrade target RNAs in the cytoplasm [85]. The possibility that ZCCHC3 also may do so is worthy of further investigation.

## Discussion

To date, scores of cellular proteins that impinge on the ability of retrotransposons to replicate in mammalian cells have been identified, and multiple mechanisms have been proposed for their action (reviewed in [5, 6, 89, 90]). Here we investigated ZCCHC3, a zinc-knuckle protein recently linked with the immune system of the cell and its action against infectious RNA viruses [19–21]. Extending previous reports [18, 22], we showed that this protein interacts with the LINE-1 ORF1 RNP and inhibits L1 and IAP retrotransposon mobility in cell culture assays. ZCCHC3 protein is known to bind viral dsDNA and dsRNA [20] and we now show that L1 RNA, and likely other cellular RNAs, are also in complex with ZCCHC3.

While this paper was under revision, a new publication confirmed that exogenously expressed ZCCHC3 protein and L1 RNPs associate in an RNA-dependent manner, and that ZCCHC3 inhibits LINE-1 cell culture retrotransposition and modulates levels of L1 RNA and ORF1p expressed exogenously in cells [91]. The authors also determined that the ZCCHC3 zinc finger domain is essential for retrotransposition, while we found its effect to be modest (Fig 2C). Notably, we introduced seven point mutations to critical residues of the zinc finger domain of the full-length protein, while Zhang et al. [91] deleted 103 amino acids of its C-terminus, perhaps compromising structural integrity of the truncated mutant protein; this could account for the discrepancy of our findings.

Both studies queried the effects of overexpression or KD of ZCCHC3 protein on L1 RNA levels in cells, but with contradictory results. Zhang et al. [91] reported reduction and increase of exogenously expressed L1 RNA, respectively, while we detected no significant change in levels of endogenous L1 RNA by RT-qPCR. In their protocol, Zhang et al. cotransfected ZCCHC3-expressing plasmids or siRNAs against ZCCHC3 with pWA367, a construct with CAG promoter driving expression of a full-length L1 tagged with a firefly luciferase gene retrotransposition reporter cassette [92]. Exogenous L1 RNA levels were assayed by RT-qPCR of cassette RNA lacking the intron following splicing.

Potential pitfalls exist for both strategies. In the case of RT-qPCR for detection of endogenous L1 mRNA levels, high L1 copy number and embedment of some L1s in longer gene transcripts could lead to misinterpretation. For example, an apparent change in what is assumed to be L1-promoter driven mRNA expression might instead be a consequence of altered expression of a gene in which targeted L1 sequence resides. On the other hand, the strategy of assaying transcription of an ectopically expressed L1 reporter construct with exogenous promoter and reporter cassette is artificial. It is also not suitable if testing effect of a protein implicated in RNA splicing, as are four members of the 25-member ZCCHC superfamily (although no role for ZCCHC3 in splicing is known) [93]. Moreover, antisense transcription of the reporter cassette from its own promoter can modify L1 RNA levels through RNAi effects [94]. RNA-Seq with expression analysis of individual intergenic full-length L1Hs elements is a more sophisticated strategy, but was beyond the scope of this paper [95–97]

ORF1p forms condensates both in vitro and in cultured cells [98, 99], and point mutations of selected basic residues reduce condensation and cytoplasmic granule formation (although for altered R261, a recent study [99] reported complete loss of granule formation, while we observed modest attenuation in puncta formation of tagged ORF1p, but with significant changes to their morphology; notably, altering residue R159 inhibited granule formation to greater degree and abolished cell culture retrotransposition) [55, 61].

Here we demonstrated that both endogenous and exogenously expressed ZCCHC3 behaves as a SG protein in stressed but not unstressed cells of multiple cell lines and forms dense cytoplasmic aggregates that colocalize with those of ORF1p. However, unlike ZCCHC3, endogenous ORF1p can form aggregates even in unstressed cells of certain cell lines. Indeed, these aggregates may not all be SGs, suggested in part by the fact that, unlike SGs, some endogenous ORF1p granules do not dissemble during cell mitosis [61, 100]. The fact that overexpressed ORF1p (unlike ZCCHC3) forms granules in G3BP1/2 DKO U2OS cells (Fig 5E) further suggests that some ORF1p granules are not SGs. Some non-SGs may be P-bodies (PBs), cytoplasmic granules that harbor molecules involved in mRNA decay and translation inhibition [57, 101, 102], although in unstressed cells endogenous ORF1p aggregates tend to abut but not overlap PBs [55, 61] (although there are reports of endogenous L1 RNA in PBs and enrichment of PB RNAs in ORF1p RNPs [42, 103]). De Luca et al. [104] recently identified ORF1p granules in Mael^-/-^ mouse male germ cells having structural and component differences from SGs or PBs and termed these L1 bodies (LBs). Other candidate cytoplasmic granules that conceivably could contain ORF1p include aggresomes, snRNP U bodies [105], or perhaps exosome granules; the RNA exosome and some of its cofactors have been reported in cytoplasmic granular structures distinct from PBs and SGs [106–108]. The dynamics of L1 ORF1p localization require further investigation.

While predominantly a cytoplasmic protein, some ORF1p may also be found in the nucleus. We reported that, in addition to colocalizing with nucleoli, in certain cell lines ORF1p also forms nuclear foci that are in part perinucleolar and also colocalize with exogenously expressed Alu RNAs [61, 109] (which in turn partially juxtapose with nucleolar-associated Cajal bodies [62], an observation bolstered by the subsequent discovery of AluACA H/ACA small RNAs [110, 111]). Formation of nuclear foci by ORF1p was recently confirmed elsewhere [99]. Mita et al. [112] reported ORF1p nuclear localization to be cell cycle-dependent, occurring mainly during mitosis and continuing into G1 phase: however, we failed to find this stringent cell cycle control of ORF1p nucleolar localization in 2102Ep cells [61]. In the present study, we detected no colocalization of ORF1p with ZCCHC3 protein in nuclear foci.

Using direct co-IP, IF, and gradient centrifugation, we linked ZCCHC3 with subunits of the RNA exosome complex (Figs 7 and 8). Notably, ZCCHC3 protein binds EXOSC5, a non-catalytic component of the exosome core, and weakly DIS3L, a cytoplasm-specific catalytic component having 3’ to 5’ exoribonuclease activity. Multiple studies have linked the exosome with modulation of retrotransposon activity. For example, Yamanaka et al. [33] reported sense and antisense transcripts of retroelements to be upregulated in rrp6−/− (EXOSC10 in humans) Drosophila larvae. LINE-1 RNAs are bound by the exosome, and both L1 RNA and ORF1p levels increase in hESCs depleted of exosomes by EXOSC3 shRNA KD [34]. Furthermore, the nuclear exosome NEXT complex targets LINE-1 and LTR RNAs in mESCs, and LINE-1 RNA decay is mediated by ZCCHC8, a subunit of that complex [35]. ZCCHC8 through its interaction with human silencing hub (HUSH) component MPP8 recruits the exosome-associated NEXT complex to MPP8-bound TE loci and transcriptionally represses L1s and retroviruses in the genome through histone H3 lysine 9 trimethylation [36, 113–116].

Like ZCCHC3 (Fig 7C), ZAP also directly binds EXOSC5 protein [29, 85], and Lee et al. [117] reported that ectopic expression of ZAP in 293T cells recruits EXOSC5 protein together with viral RNA from the cytosol to RNA granules (reviewed in [118, 119]). We hypothesize for future investigations that ZCCHC3 acts as a bridging protein for associated components of the cytoplasmic exosome, an interaction that could involve EXOSC5 and that perhaps also includes ZAP.

Indeed, we discovered an association between ZCCHC3 and the better-characterized interferon-stimulated gene products MOV10 and ZAP, host proteins known to restrict viral infection in mammals and previously shown by ourselves and others to potently inhibit cell culture retrotransposition [27–32]. MOV10 also associates with the RNA-induced silencing complex (RISC) in PBs and SGs where it regulates miRNA-mediated gene repression of target mRNAs (reviewed in [120, 121]). All three proteins associate with each other in direct co-IP experiments and colocalize together in cytoplasmic granules (Fig 6). We suggest that ZCCHC3 and ZAP, and perhaps MOV10, may be linked in the same anti-viral/anti-retrotransposon complex.

## Materials and methods

### Plasmids, and RNAi constructs, and cell lines

Clones obtained as gifts included IAP-neo^TNF^ (M. Dewannieux, Institut Gustave Roussy, Villejuif [48]), shN3 and shEXOSC3 shRNAs cloned in DNA transposon piggyBac vector (S. Wolin and S. Sim, NIH, MD [34]), C-terminal HA-tagged SKIV2L (SKIC2) and TTC37 (SKIC3) cloned in pCAGGS-HA vector (M. Frieman, University of Maryland School of Medicine, Baltimore [78]), EGFP-tagged HBS1LV3 which we recloned in pcDNA6 myc/his B with C-terminal V5-tag (A. Dziembowski, Warsaw University, Poland [83]), pFLAG-MOV10 (FL-MOV10) (R. Burdick, National Cancer Institute, NIH [122]), GFP-PARP13.2 (GFP-ZAP-S) (A. Leung, Johns Hopkins School of Medicine, Baltimore), ZCCHC3-shRNA-2 (targeting sequence: 5’-AGTACAAATGCGAGATCGA-3’) and ZCCHC3-shRNA-4 (5’-GGAGCAAGTCCAGCTTGAA-3’) cloned in pSuper-retro vector (H.-B. Shu, Wuhan University, China [20]), CONT shRNA-16 cloned in pLKO.1-TRC cloning vector [46] (5’-GAGCACTCTGAACTACCTGTTCAAGAGACAGGTAGTTCAGAGTGCTCTTTT-3’, A. Long, Johns Hopkins School of Medicine, Baltimore), and pc5CFLAG-Z7 (FL-ZCCHC7) cloned in pcDNA5/FRT/TO vector (T. Jensen, Aarhus University, Denmark [80]).

Ultimate ORF cDNAs (Invitrogen) were cloned with V5-epitope tags and tobacco etch virus (TEV) protease cleavage sites on their N-termini by shuttling them from pENTR221 vector into pcDNA3.1/nV5-DEST vector using Gateway Technology (Invitrogen). cDNAs and their clone ID numbers were DIS3 (EXOSC11) (IOH29073), DIS3L (isoform 2) (IOH13442), EXOSC10 (RRP6) (IOH26102), EXOSC3 (RRP40) (IOH4246), EXOSC5 (RRP46) (IOH6517), EXOSC7 (RRP42) (IOH12658), EXOSC8 (RRP43) (IOH10049), MOV10 (IOH4005), TRIM25 (IOH11168), WDR61 (SKIC8) (IOH12997), and ZCCHC3 (IOH40787). Control vectors pcDNA5 FRT/TO (pcDNA5), pcDNA6 myc/his B (pcDNA6), and pcDNA3.1/nV5-DEST were from Thermo Fisher Scientific.

PCR amplification of V5-TEV-ZCCHC3 was used to generate FLAG-tagged ZCCHC3-FL and PCR QuickChange mutagenesis was used to generate ZCCHC3-FL mutant constructs ZCCHC3-3AA (C338,356,375A), ZCCHC3-FL-7AA (C353,366,372,375A/H380A/CP385,386AA), ZCCHC3-FL-Δ55–210, and ZCCHC3-FL-S363A. The following clones were previously described: 99-PUR-RPS-EGFP, 99-PUR-JM111-RPS-EGFP [45], pc-L1-1FH, pc-L1-RP [28], ORF1-EGFP-L1-RP [62], and ZAP-L-FL [29].

Human 2102Ep embryonal carcinoma (a gift from P.K. Andrews, University of Sheffield), embryonic kidney (HEK) 293T (ATCC), cervical cancer HeLa-JVM [49], and osteosarcoma wild-type U2OS-WT and G3BP1/2 U2OS-DKO (a gift from N. Kedersha, Harvard, MA [59]) cell lines were grown in Dulbecco’s modified Eagle’s medium (Invitrogen) supplemented with 10% FBS (Hyclone), GlutaMax, and Pen-Strep (Invitrogen). Cell transfection reagents were FuGENE HD or FuGENE 6 (Promega). Stress granules were induced in cells by 0.25 mM sodium arsenite incubated for 50 min.

To generate clonal 293T ZAP KO cell lines, the following reagents were purchased from Integrated DNA Technologies: Alt-R S.p. Cas9 Nuclease V3, Alt-R CRISPR-Cas9 tracrRNA ATTO 550, and the Alt-R CRISPR-Cas9 crRNA sequences,

Hs.Cas9.ZC3HAV1.1.AB GCACGGGCUGAACCCCGACGGUUUUAGAGCUAUGCU and

Hs.Cas9.ZC3HAV1.1.AE GAGUAGAGAUCGGUUCUUUCGUUUUAGAGCUAUGCU.

Forty hours post-transfection, single cells were isolated by FACS into 96-well plates, monitored microscopically for red-fluorescence and single colony formation, expanded, and frozen. Names of clonal lines beginning with AB used Alt-R CRISPR-Cas9 crRNA sequence Hs.Cas9.ZC3HAV1.1.AB and those beginning with AE used Hs.Cas9.ZC3HAV1.1.AE (Figs 2E and 6H).

### Antibody analyses, microscopy, and protein analyses

Commercial antibodies included rabbit (rb) α-SKIV2L2 (MTR4) (A13258), rb α-ZCCHC3 (A17235; α-ZCCHC3-Ab) (Abclonal), rb α-ZCCHC3 (ARP50730_P050; α-ZCCHC3-Av) (Aviva Systems Biology), mouse (ms) α-DYKDDDDK (binds FLAG-tag, 9A3), rb α-DYKDDDDK (D6W5B), ms α-HA-tag (6E2), rb α-HSP90 (#4874), and rb α-ZCCHC3 (#65321; α-ZCCHC3-CS) (Cell Signaling Technology), ms α-FLAG-M2 (F1804/F3165) (MilliporeSigma), ms α-DIS3 (PCRP-DIS3-1A7), ms α-G3BP2 (PCRP-G3BP2-1C7) (Deveopmental Studies Hybridoma Bank), rb α-EXOSC3 (15062-1-AP), rb α-MOV10 (10370-1-AP), rb α-alpha tubulin (11224-1-AP), rb α-ZC3HAV1 (16820-1-AP, α-ZAP) (ProteinTech), ms α-V5-tag (Thermo Fisher Scientific), goat (gt) α-eIF3η (N-20), and gt α-TIA1 (C-20) (Santa Cruz Biotechnology). Monoclonal ms α-ORF1-4H1 was provided by K. Burns (Dana-Farber Cancer Institute, Boston, also available as Millipore Sigma MABC1152 [38]) and rb polyclonal α-L1Hs-ORF1p by O. Weichenrieder (Max-Planck Institute, Tubingen). Donkey Cy3-, DyLight 488- or DyLight 549-conjugated and HRP-conjugated secondary antibodies were from Jackson ImmunoResearch Laboratories, or MilliporeSigma in the case of Figs 1D, 1F, 6B and 7D.

Western blotting, IP, and IF were performed as previously described [28, 29, 55, 62]. Western blots were run on NuPAGE 4–12% Bis-Tris gels (Fisher Scientific). The protein markers used for all Westerns of this study were Novex Sharp Pre-stained Protein Standard (Fisher Scientific), except for those of Figs 1D, 1F, 6B and 7D which used Precision Plus Protein Dual Color Standards (Bio-Rad). IF-stained cells were examined using a Nikon Eclipse Ti-A1 confocal microscope with NIS-Elements AR software.

MS sequencing and database analyses were performed by the Johns Hopkins Mass Spectrometry and Proteomics Facility as previously described [29, 40]. Post-translational modifications (including phosphorylation and gly-gly modifiation on lysines) of ZCCHC3 protein were annotated with Byonic v3.6.0 software (Protein Metrics Inc.),

### Retrotransposition assay

The EGFP L1 cell culture retrotransposition assay was conducted as previously described [28, 29, 45]. A retrotransposition-defective L1 construct, 99-PUR-JM111-EGFP, with ORF1 mutation was used as negative control and for flow cytometry gating. The IAP retrotransposition assay was conducted as described [48, 61]. Empty vectors used for controls for the L1 retrotransposition assay were pcDNA6 myc/hisB or pcDNA3 (Thermo Fisher Scientific).

### Protein isolation and immunoprecipitation

For most co-IP experiments with FLAG-tagged constructs, HEK 293T cells in T75 flasks were transfected with a total of 15 μg of test plasmid and/or empty vector and expanded for approximately 45 h, followed by whole cell lysate preparation and homogenization by Diagenode Bioruptor. Treatment of samples with 25 μg/ml DNase-free RNase (Roche) and 25 μg/ml RNaseA (Qiagen) was conducted in the absence of RNase inhibitors. Lysate preparation, IP, and sample recovery were as previously described using α-FLAG-M2 Magnetic Beads (MilliporeSigma) and 3XFLAG Peptide (Sigma-Aldrich) for elution through Corning Costar Spin-X centrifuge tube filters [29, 75]. Eluted samples were combined with 3XSDS loading buffer/phenylmethylsulfonyl fluoride (PMSF) and heated at 95°C for 5 min prior to Western blotting analyses.

Selected experiments (Figs 1D, 1F, 6B and 7D) involving co-IP of endogenous proteins were performed using HEK 293T cells grown in T75 flasks (for endogenous ORF1p and MOV10 IPs, Figs 1F and 6B) or 10 cm tissue culture plates. Cells were transfected with Lipofectamine 2000 (ThermoFisher) and 6 μg of V5-TEV-EXOSC5 or empty vector (pcDNA3.1/nV5-DEST) for Figs 1D and 7D. Conditions for IP were as described below for RNA extraction and IP (RIP). Forty-eight hours after transfection, whole cell lysates were prepared and incubated with Protein G Dynabeads (Fisher) and bound α-ZCCHC3-CS antibody (1:10 dilution) for 3 h at 4°C with rotation. After washes, proteins were eluted from the beads with NuPage LDS sample buffer (Thermo Fisher) and dithiothreitol (DTT) and heated at 70°C for 20 min for Western blotting analyses.

### RNA extraction and RT-qPCR

2×10^6^ HEK 293T cells were transfected in 10-cm tissue culture plates with 6 μg of ZAP-L-FL, FL-MOV10, ZCCHC3-FL or ZCCHC3-FL-7AA) using Lipofectamine 2000. Transfection with pcDNA6/myc-His B (empty vector) was used as a negative control for the IP. RIP and RNA extraction was performed according to [52]. Following IP of FLAG-tagged proteins on Protein G Dynabeads (Thermo Fisher) and α-FLAG-M2 (Millipore Sigma, F3165) for 3 h at 4°C with rotation and 5 washes with lysis buffer, 10% of sample-beads were used for protein extraction and Western blotting by adding LDS sample buffer and DTT and heating the samples at 70°C for 20 min. 90% of the sample beads were incubated with RQ1 DNAse for 30 min for later RNA extraction with Trizol LS (Ambion). One μg of RNA was subsequently retreated with RQ1 DNAse and purified by phenol/chloroform extraction. cDNA was synthesized using High-Capacity cDNA Reverse Transcription Kit (Applied Biosystems), followed by qPCR (GoTaq qPCR Mix, Promega) in triplicate for each sample following the manufacturer’s instructions. No-RT and no-template controls were used to verify the absence of contaminating genomic DNA. RT-qPCR cycling parameters were as follows: 10 min at 95°C, 40 cycles of 15 sec at 95°C, followed by 60 sec at 60°C. Endogenous L1 mRNA was quantified using the following primers.

5UTR L1Hs-Fwd: CGCAGGCCAGTGTGTGT

5UTR L1Hs-Rev: TCACCCCTTTCTTTGACTC

N51-Fwd (ORF1): GAATGATTTTGACGAGCTGAGAGAA

N51-Rev (ORF1): GTCCTCCCGTAGCTCAGAGTAATT

N22-Fwd (ORF2): CAAACACCGCATATTCTCACTCA

N22-Rev (ORF2): CTTCCTGTGTCCATGTGATCTCA

Other primer pairs included:

β-actin-Fwd: ACCGAGCGCGGCTACAG

β-actin-Rev: CTTAATGTCACGCACGATTTCC

GAPDH-Fwd: TGCACCACCAACTGCTTAGC

GAPDH-Rev: GGCATGGACTGTGGTCATGAG

Primers were validated to perform at >90% efficiency in the RT-qPCR assay. Melting curve analysis confirmed the identity of the amplified products. For the IP experiment of Fig 1E, the Relative Standard Curve Method was used to quantify RNA levels, as described by the suppliers. Experiments of Fig 3B using the ΔΔCt method for RNA quantification, normalizing to GAPDH RNA [124].

### Assessment of toxicity

Trypan Blue exclusion assays were performed in HEK 293T cells at 4 days post-transfection. Following staining, live and dead cells were counted using a Countess II Automated Cell Counter (Thermo Fisher Scientific). Use of the MultiTox-Fluor Multiplex Cytotoxicity Assay kit (Promega) followed manufacturer’s instructions. This assay simultaneously measures cell viability and cytotoxicity in a single-reagent reaction permitting ratios of live to dead cell readings to be calculated.

### Velocity sedimentation centrifugation

Discontinuous sucrose density gradients at concentrations of 50%, 40%, 30%, 20%, and 10% were generated in 14 ml Beckman Coulter Ultra-Clear Open-Top tubes according to the method of [85]. Each layer was frozen at -80 C prior to adding the next. The cell lysis buffer was 50 mM Tris-HCl pH 7.5, 150 mM NaCl, 0.5% NP40, 1 mM DTT, 10 μM zinc, RNasin, vanadyl ribonucleoside complexes, and protease inhibitors. 400 μl of 293T whole cell extracts were prepared, drawn through 18 and 25-gauge hypodermic needles, clarified by centrifugation for 10 min at 13,000 rpm, and loaded on sucrose gradients. Centrifugation was performed in a Beckman Coulter SW 41Ti swinging bucket rotor at 4°C for 3 h at 38,000 rpm. 900 μl fractions were collected from the top of the tube with a hypodermic needle and syringe, and 25 μl of each fraction were loaded in each well of Western gels.
